# Proximate composition, fatty acid characteristics, and amino acid profile of European anchovy (*Engraulis encrasicolus* L. 1758): monthly and seasonal influences

**DOI:** 10.3389/fnut.2025.1684958

**Published:** 2025-10-13

**Authors:** Barış Karslı, Özen Yusuf Öğretmen, Muhammet Emanet

**Affiliations:** ^**1**^Faculty of Fisheries, Department of Seafood Processing Technology, Recep Tayyip Erdogan University, Rize, Türkiye; ^**2**^Faculty of Fisheries, Department of Fisheries, Recep Tayyip Erdogan University, Rize, Türkiye

**Keywords:** anchovy, nutritional value, proximate composition, amino acids, fatty acids

## Abstract

This study investigated the proximate composition, amino acid, and fatty acid profiles of anchovy (*Engraulis encrasicolus*) collected from the Southeastern Black Sea on both monthly and seasonal bases. Crude protein (17.65–20.24%), crude fat (5.29–14.51%), moisture (64.61–73.12%), and crude ash (1.30–1.82%) contents showed significant fluctuations. Palmitic acid was the dominant saturated fatty acid (16.97–20.89%). Polyunsaturated fatty acids (PUFAs) reached their highest values in summer (33.50%) and June (35.30%), while the lowest levels were detected in autumn (28.76%) and November (27.14%). Eicosapentaenoic acid (EPA) peaked in spring and May (up to 8.95%) and was lowest in autumn and September (6.47%). Docosahexaenoic acid (DHA) was highest in summer and September (19.86%) and lowest in autumn and November (13.78%). The atherogenicity and thrombogenicity indices varied between 0.77–0.73 and 0.81–0.69 seasonally, and 0.35–0.90 and 0.36–0.27 monthly, respectively. Total essential amino acids ranged from 8,727 to 10,039 mg/100 g, while total non-essential amino acids ranged from 7,961 to 8,894 mg/100 g. Lysine was the most abundant essential amino acid, whereas glutamic acid was the predominant non-essential amino acid.

## 1 Introduction

Fishery products are widely regarded as healthy foods because they are rich in omega-3 polyunsaturated fatty acids (PUFAs) and their ability to provide various essential nutrients (1, 2). Recently, fish has received significant attention for its ability to satisfy a substantial portion of people’s daily nutritional needs. Extensive research shows that fish consumption can help protect against cardiovascular diseases, obesity, inflammation, diabetes, and cancer due to its high levels of PUFAs, essential minerals, and protein (3, 4). Multiple studies have examined the link between n-3 fatty acid intake and fish consumption, emphasizing their positive effects on cognitive function and overall well-being in humans (5, 6). High-quality proteins are easily digestible and rich in essential amino acids (EAA), for which fish constitute an excellent source, particularly providing valine, leucine, isoleucine, threonine, phenylalanine, lysine, and tryptophan (7, 8). Additionally, fish proteins contribute to health by supporting bodily functions and reducing the risks of metabolic syndromes, inflammation, and insulin resistance (2). The quality of fish is evaluated by considering factors such as color, smell, and nutritional composition (9). Fresh fish consists of 66–81% moisture, 16–21% protein, 1.2–1.5% minerals, 0.2–25% fat, and 0–0.5% carbohydrates (10). In addition, the chemical composition of the fish varies according to age, sex, environment, feed intake, and species (11). The proximate composition of small pelagic fish such as anchovies may vary depending on the fishing season (12). Plankton-feeding species such as anchovy naturally experience seasonal differences in their proximate composition since plankton production depends on the season (13). Anchovy is a fish species rich in protein, PUFAs, and phospholipids (14). Classified as an oily fish because of its high fatty acid content, anchovy is widely consumed for its richness in PUFAs (52).

*Engraulis encrasicolus,* commonly known as the European anchovy (Linnaeus, 1758), belongs to the order Clupeiformes and the family *Engraulidae*, which encompasses 18 genera and 181 species (15). This species constitutes approximately 50% of the pelagic fish population in the Mediterranean (52). In Turkey, anchovy is primarily harvested using purse seines. In 2023, approximately 274,000 tons of anchovies were caught in Türkiye, representing 70.8% of the country’s total marine fish catch (16). Anchovy fishing along the Black Sea coast is an important source of both economic income and nutritional value, especially for local communities (17). Previous studies on the nutritional composition, amino acid content, and fatty acid profile of anchovy (*Engraulis encrasicolus*) (8, 18–23) have made significant contributions to understanding the nutritional properties of this species. However, most of these studies have limited their sampling to the fishing season, typically between September and April, and have often focused on samples collected during a specific month or a short period. This methodological approach does not allow for a comprehensive assessment of seasonal variations and may overlook potential fluctuations in the nutritional content of the species throughout the year. In contrast, the present study is the first to systematically and continuously examine the proximate composition, fatty acid profile, and amino acid content of anchovies over 12 months. In this respect, the study provides a comprehensive overview of the seasonal changes in the nutritional characteristics of the species across the entire year, rather than restricted to particular months. Thus, this study contributes a more holistic and dynamic dataset that addresses the limitations of previous research.

## 2 Materials and methods

### 2.1 Chemicals and reagents

All reagents and solvents used in this study were of analytical grade and purchased from Sigma–Aldrich GmbH (Steinheim, Germany). The instruments utilized in the analyses included a hot air oven (Nüve ES-120, Türkiye), a muffle furnace (Şimşek Loborteknik, Türkiye), a solvent extraction unit (Velp SER 148/6, Milano, Italy), a Kjeldahl digestion unit (Behr Inkjel M, Dusseldorf, Germany), a Soxhlet apparatus (Bher S5, Dusseldorf, Germany), an electronic balance (AND GR-200, Japan), and an amino acid analyzer (Shimadzu Ultra-Fast Liquid Chromatography, Japan).

### 2.2 Collection of anchovies

**Figure 1:**
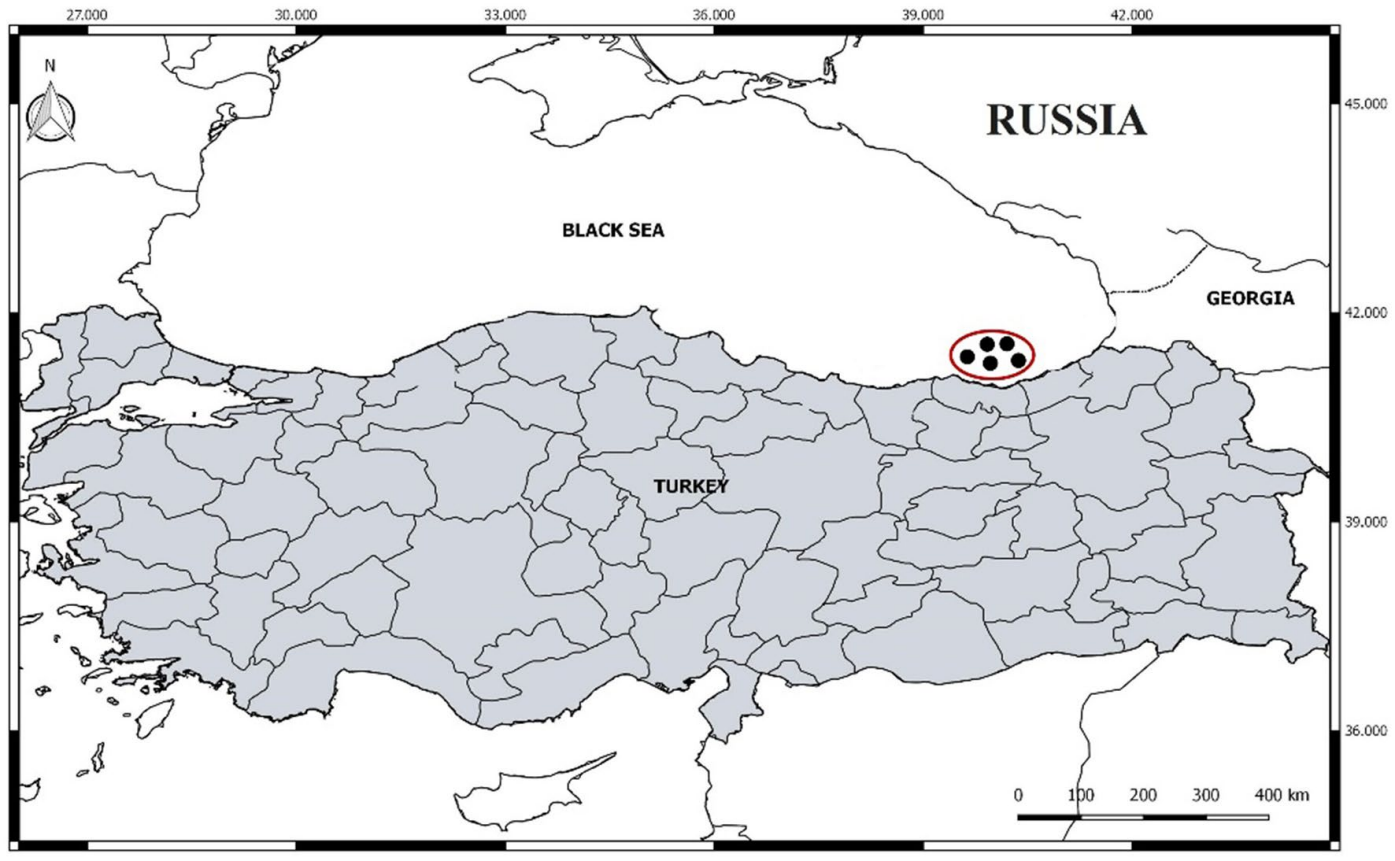
Sampling area of anchovies from the Southeastern Black Sea.

Anchovy samples (*Engraulis encrasicolus* Linnaeus, 1758) were collected monthly from the Southeastern Black Sea, specifically from the coastal region of Trabzon, Türkiye, between September 2021 and August 2022. During the legal fishing season (September to April), samples were acquired through commercial purse-seine operations. In the off-season (May to August), specimens were obtained as bycatch from local fishing cooperatives and artisanal coastal fishers active in the area (Figure 1). Approximately 5 kg of anchovy were provided each month. Seasonal samples were acquired by blending equal amounts of anchovies collected in the months representing each season (Autumn: September, October, November; Winter: December, January, February; Spring: March, April, May; Summer: June, July, August). No distinction was made between age and gender when providing samples. Only anchovies over the legal catch length of 9 cm were selected.

All anchovy samples were commercially caught, already dead at the time of acquisition, and handled in accordance with national fishing regulations. *Engraulis encrasicolus* is not a protected or endangered species (IUCN Red List: Least Concern). Therefore, no approval from an institutional animal ethics committee was required for this study.

The fish provided were placed in polystyrene boxes filled with ice and delivered to the laboratory within an hour. Biometric measurements, including the length and weight of each fish, were recorded, and standard deviations (SD) were calculated. Anchovy samples were then stored in a deep freezer at −20 °C until further analysis.

### 2.3 Sample preparation

Frozen fish were thawed at a temperature of 4 ± 2 °C prior to analysis. The heads, internal organs, and bones were then removed. For proximate analysis, the processed fish were dried in a hot air oven set at 105 °C until constant weight (≈24 h) and subsequently homogenized using a grinder. Samples designated for amino acid analysis were filleted and transported fresh to the TÜBİTAK-MAM Food Institute (Gebze, Turkey).

### 2.4 Proximate analysis

Monthly proximate analyses of anchovy samples were carried out following the procedures outlined in the AOAC methods ((24); methods 981.10, 922.06, 923.03, and 925.10). Crude protein content was calculated as total nitrogen × 6.25, after determining total nitrogen by the Kjeldahl method. The fat content was measured using the Soxhlet Avanti automatic system with petroleum ether as the solvent. Ash content was determined by burning the samples in a muffle furnace at a temperature of 550 °C until a constant weight was achieved. To measure moisture content, the samples were dried in a universal oven at 105 °C until they reached a constant weight. All analyses were performed in triplicate.

### 2.5 Fatty acid profile analyses

Fatty acid methyl esters (FAMEs) were analyzed as described by Öğretmen (8). Fatty acid methyl esters were acquired through a transmethylation process. Initially, 10 mg of the extracted oil was dissolved in 2 mL of n-hexane (Sigma-Aldrich, Steinheim, Germany). Then, 4 mL of 2 M methanolic potassium hydroxide (KOH) (Merck, Darmstadt, Germany) was added to the mixture. The solution was vortexed for 2 min at room temperature to ensure thorough mixing. Following this, the mixture was centrifuged for 10 min, and the upper hexane (1 mL) layer containing the methyl esters was collected for gas chromatographic analysis. The fatty acid composition was determined using gas chromatography–mass spectrometry (GC–MS) (QP2010 Ultra with AOC-20i + s model autosampler) using a mass selective detector (GC–MS QP 2010 PLUS) equipped with the GC/MS solutions software (Shimadzu, Kyoto, Japan). The absolute fatty acid content per 100 g was calculated based on the method described by Greenfield and Southgate (25), applying a fatty fish conversion factor of 0.90. All measurements were carried out in triplicate, and the outcomes were reported as mean values ± standard deviation, expressed as a percentage of the total GC peak area.

The nutritional value of the lipids was assessed using several indices, including the atherogenic index (AI) (26) (Equation 1), thrombogenic index (TI) (26) (Equation 2), hypocholesterolemic/hypercholesterolemic ratio (h/H) (27) (Equation 3), polyene index (PI) (28) (Equation 4), and fish lipid quality (FLQ) (29) (Equation 5). These indices were calculated according to the following equations:


(1) $$\mathrm{AI}=\frac{C12:0+4.C14:0+C16:0}{\sum \text{MUFA}+\sum n3+\sum n6}$$

(2) $$\mathrm{TI}=\frac{C14:0+C16:0+C18:0}{0.5.\sum \text{MUFA}+0.5.\sum n6+3.\sum n3+\sum n3/\sum n6}$$

(3) $$h/H=\frac{C18:1+C18:2+C18:3++C20:1+C22:1+C24:1}{C14:0+C16:0}$$

(4) $$\mathrm{PI}=\frac{\mathrm{EPA}+\mathrm{DHA}}{C16:2}$$

(5) $$\mathrm{FLQ}=\frac{\left(\mathrm{EPA}+\mathrm{DHA}\right)}{\sum \mathrm{FA}}x\;100$$

Where ∑MUFA = total monounsaturated fatty acids; n-3 = omega-3 PUFA; n-6 = omega-6 PUFA; eicosapentaenoic acid (EPA) = relative concentration of EPA in the total FAME (%); docosahexaenoic acid (DHA) = relative concentration of DHA in the total FAME (%).

### 2.6 Amino acid composition

Amino acid composition analysis of the fish samples was conducted at the TUBITAK-MAM Food Institute. The analytical method was developed in-house by modifying procedures previously described by Dimova (53) and Gheshlaghi et al. (56). Total amino acid content was quantified using a Shimadzu Ultra-Fast Liquid Chromatography (UFLC) system equipped with a UV detector. This approach involved hydrolyzing the proteins in the sample to release amino acids, followed by derivatization with phenyl isothiocyanate, and subsequent detection via UFLC-UV. A total of 16 amino acids were quantified in milligrams per 100 grams (mg/100 g), including aspartic acid, glutamic acid, serine, glycine, arginine, histidine, threonine, lysine, alanine, proline, leucine, isoleucine, tyrosine, phenylalanine, valine, and methionine. For calibration, an amino acid standard solution (AAS 18; 1–105 μg/μL) was obtained from Sigma-Aldrich, Chemie GmbH, Munich, Germany.

### 2.7 Statistical analysis

All analyses were performed in triplicate. A one-way analysis of variance (ANOVA) followed by Tukey’s post-hoc test was used to assess significant differences in proximate composition, fatty acid profile, and amino acid composition among months and seasons. Statistical significance was considered at *p* < 0.05. All statistical analyses were conducted using JMP Pro 13 (SAS Institute, Cary, NC, USA). Results are expressed as mean ± standard deviation (SD).

## 3 Results and discussion

**Figure 2:**
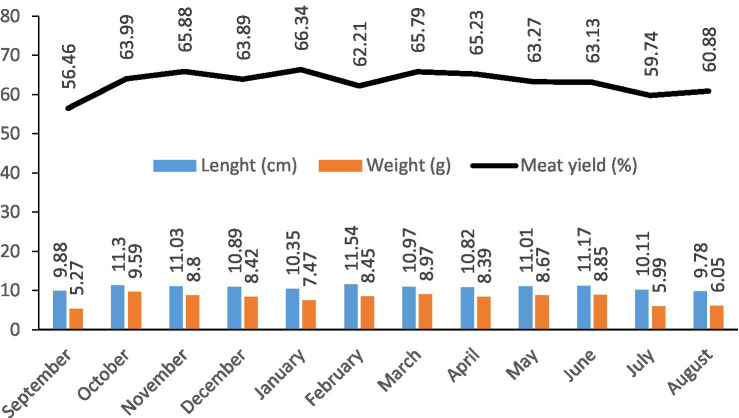
Seasonal length, weight and meat yield of anchovies from the Southeastern Black Sea.

A total of 60 kg of anchovy samples were used in the study. The mean length and weight of the anchovies were 10.73 ± 0.54 cm and 7.91 ± 1.33 g, respectively. The mean meat yield obtained from muscle tissues was 63.06 ± 2.77% (Figure 2).

### 3.1 Changes in proximate composition

**Figure 3:**
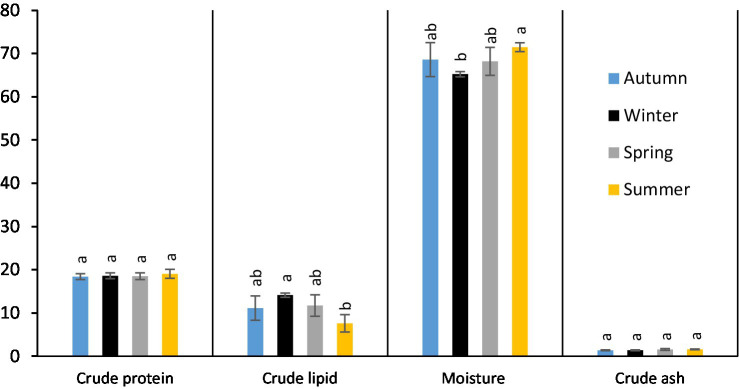
Seasonal proximate composition of anchovies from the Southeastern Black Sea (%). Different letters indicate significant statistical differences in the proximate composition between seasons (*p* < 0.05).

Monthly and seasonal variations in the proximate composition of anchovy (*E. encrasicolus*) muscle fillets are presented in Tables 1, 2 and Figure 3, respectively. The lowest crude protein content was observed in September (17.65%), while the highest was recorded in August (20.24%). The protein content fluctuated throughout the year, with a significant absolute difference of 2.59 percentage points (*p* < 0.05). According to seasonal data, the highest protein content occurred in summer (19.06%) and the lowest in autumn (18.41%), though no significant seasonal differences were found (*p* > 0.05). The crude fat content in anchovy fillets was highest in January (14.51%) and lowest in August (5.29%). Monthly analysis demonstrated a consistent decrease in crude fat content from February to August, followed by an increase from September to January. Throughout the year, a statistically significant absolute difference of 9.22 percentage points (*p* < 0.05) was observed in crude fat content. The highest crude fat content was found in fillets collected during the winter (14.15%), followed by spring, autumn, and summer. The difference between the highest and lowest lipid values was significant at an absolute 6.54 percentage points (*p* < 0.05). Moisture content in the anchovy fillets ranged from 64.61% in January to 73.12% in September, with a significant absolute difference of 8.51 percentage points (*p* < 0.05). Moisture content was consistently above 70% between May and September and fell below 70% from October to April, showing significant monthly variations (*p* < 0.05). These fluctuations may be associated with environmental factors such as water temperature, reproductive activity, and feeding intensity. The lower values observed in autumn and winter were likely related to increased fat deposition, as moisture is inversely correlated with crude fat contents. However, when data were averaged by season, no significant differences were detected (*p* > 0.05). The highest moisture rate was recorded in summer (71.45%), followed by autumn (68.59%), spring (68.20%), and winter (65.23%) seasons; the difference in moisture amounts between seasons was not found to be statistically significant (*p* > 0.05). Ash content was highest in May (1.82%) and lowest in January (1.30%), showing a significant absolute difference of 0.52 percentage points (*p* < 0.05). Seasonally, the highest ash content was observed in summer with 1.62%, followed by spring, winter, and autumn. The seasonal absolute difference of 0.23 percentage points was not significant (*p* > 0.05).

**Table 1:** Monthly proximate composition of anchovies from the southeastern Black Sea (%).

Sampling time		Crude protein	Crude lipid	Moisture	Crude ash
2021	September	17.65 ± 0.11^c^	7.93 ± 0.09^e^	73.12 ± 0.64^d^	1.31 ± 0.10^d^
	October	18.89 ± 0.98^bc^	12.50 ± 0.61^c^	66.10 ± 1.57^ab^	1.36 ± 0.11^d^
	November	18.71 ± 0.25^bc^	13.03 ± 0.17^bc^	66.57 ± 0.13^ab^	1.52 ± 0.04^bc^
	December	17.85 ± 0.14^bc^	14.36 ± 0.20^a^	65.26 ± 0.78^ab^	1.40 ± 0.07^cd^
2022	January	19.14 ± 0.09^ab^	14.51 ± 0.28^a^	64.61 ± 1.11^a^	1.30 ± 0.18^d^
	February	18.84 ± 0.06^bc^	13.59 ± 0.30^ab^	65.84 ± 0.31^ab^	1.50 ± 0.14^bc^
	March	18.76 ± 0.25^bc^	14.00 ± 0.25^ab^	65.45 ± 0.69^ab^	1.45 ± 0.06^bc^
	April	19.15 ± 0.24^ab^	12.11 ± 0.13^c^	67.38 ± 1.75^b^	1.47 ± 0.14^bc^
	May	17.65 ± 0.23^c^	9.08 ± 0.05^d^	71.77 ± 0.71^cd^	1.82 ± 0.04^a^
	June	18.34 ± 0.06^c^	8.72 ± 0.23^de^	70.60 ± 0.73^c^	1.70 ± 0.01^ab^
	July	18.60 ± 0.18^bc^	8.82 ± 0.10^de^	71.16 ± 0.84^cd^	1.50 ± 0.11^bc^
	August	20.24 ± 0.31^a^	5.29 ± 0.12^f^	72.60 ± 0.37^cd^	1.67 ± 0.08^ab^

Different superscript letters denote significant statistical differences in the monthly data within the same column (*p* < 0.05).

**Table 2:** Proximate composition of anchovy captured in different seasons (%).

Sampling time	Crude protein	Crude lipid	Moisture	Crude ash
Autumn	18.41 ± 0.67^a^	11.15 ± 2.80^ab^	68.59 ± 3.92^a^	1.39 ± 0.10^a^
Winter	18.61 ± 0.67^a^	14.15 ± 0.49^a^	65.23 ± 0.61^a^	1.40 ± 0.10^a^
Spring	18.52 ± 0.77^a^	11.73 ± 2.48^ab^	68.20 ± 3.23^a^	1.58 ± 0.20^a^
Summer	19.06 ± 1.03^a^	7.61 ± 2.00^b^	71.45 ± 1.03^a^	1.62 ± 0.10^a^

Different superscript letters denote significant statistical differences for content or percentage data within rows (one-way ANOVA. *p* < 0.05).

When compared to other previous studies, Öğretmen (8) reported that the crude protein content in anchovy fillets from the Black Sea coast ranged from 16.1 to 17.2%, which aligns with our current findings. Kari et al. (18) also reported protein contents of 16–21%, consistent with our results. In contrast, Traina et al. (2) found protein values of 19.9–24.8% in Mediterranean anchovy species, which were higher than in our study, likely due to differences in species or fishing regions. Reported fat contents of 8.23–12.22% (8) and 9.33% (19) were similar to our findings. The decline in crude fat contents from May to September in our study may be attributed to increased sea temperatures and the spawning season.

For moisture content, Albrecht-Ruiz and Salas-Maldonado (30) reported 76.48% in winter and 72.18% in autumn for *Engraulis ringens*, both higher than our results. Their ash content values (1.92–1.96%) were also higher. Similarly, Pariona-Velarde et al. (20) reported 79.2% moisture and 1.2% ash in *E. ringens*. In contrast, Gençbay and Turhan (22) observed 68.08% moisture and 1.52% ash in Black Sea anchovy, while Öğretmen (8) reported values consistent with ours. Such variability across studies may be attributed to environmental changes, diet composition, and physiological processes, such as spawning and migration, all of which influence fish muscle composition (31).

In the present study, crude fat content was highest in winter, corresponding to the pre-spawning and spawning season (December–February) and March. Anchovies utilize their nutritional reserves during this period, leading to reduced crude fat contents after March. Consequently, moisture content fluctuated inversely with fat contents throughout the year.

### 3.2 Changes in fatty acid (FA) profile

The monthly and seasonal variations in the fatty acid composition of the muscle fillet of European anchovy (*E. encrasicolus*) are summarized in Tables 3, 4.

**Table 3:** Monthly fatty acid composition and nutritional quality indexes of anchovies from the southeastern Black Sea (%).

Fatty acid (FA)	2021				2022							
	September	October	November	December	January	February	March	April	May	June	July	August
*C4:0*	**-**	**-**	**-**	**-**	0.69 ± 0.05^a^	0.08 ± 0.01^c^	0.27 ± 0.01^b^	0.29 ± 0.05^b^	0.21 ± 0.06^bc^	0.17 ± 0.04^bc^	**-**	**-**
*C12:0*	0.04 ± 0.01^b^	0.07 ± 0.02^ab^	0.06 ± 0.01^ab^	0.07 ± 0.01^ab^	0.06 ± 0.01^ab^	0.06 ± 0.01^ab^	0.08 ± 0.02^a^	0.07 ± 0.01^ab^	0.07 ± 0.01^ab^	0.06 ± 0.01^ab^	0.06 ± 0.01^ab^	0.04 ± 0.01^b^
*C13:0*	0.03 ± 0.00^a^	0.04 ± 0.01^a^	0.05 ± 0.01^a^	0.04 ± 0.00^a^	0.06 ± 0.02^a^	0.03 ± 0.00^a^	0.04 ± 0.01^a^	0.05 ± 0.00^a^	0.04 ± 0.00^a^	0.04 ± 0.01^a^	**-**	0.04 ± 0.01^a^
*C14:0*	5.37 ± 0.02^e^	6.04 ± 0.01^ab^	5.88 ± 0.01^bc^	5.93 ± 0.01^bc^	6.46 ± 0.40^a^	5.86 ± 0.08^cd^	6.52 ± 0.09^a^	6.35 ± 0.04^ab^	6.35 ± 0.03^ab^	6.22 ± 0.05^ab^	5.57 ± 0.04^d^	5.80 ± 0.05^cd^
*C15:0*	0.90 ± 0.01^c^	1.09 ± 0.04^ab^	1.06 ± 0.01^b^	1.08 ± 0.03^ab^	1.21 ± 0.08^a^	0.89 ± 0.01^e^	1.02 ± 0.02^bc^	1.12 ± 0.03^ab^	0.94 ± 0.01^bc^	0.97 ± 0.03^bc^	0.91 ± 0.01^c^	0.92 ± 0.02^c^
*C16:0*	18.12 ± 0.14^bc^	19.48 ± 0.21^ab^	19.16 ± 0.24^ab^	19.27 ± 0.22^ab^	19.52 ± 0.93^ab^	17.74 ± 0.13^c^	19.74 ± 0.16^ab^	19.69 ± 0.29^ab^	16.97 ± 0.07^d^	17.24 ± 0.22^cd^	20.89 ± 0.10^a^	18.33 ± 0.18^bc^
*C17:0*	1.09 ± 0.02^b^	1.12 ± 0.01^b^	1.10 ± 0.03^b^	1.11 ± 0.01^b^	1.09 ± 0.02^b^	0.95 ± 0.02^d^	1.01 ± 0.03^cd^	1.06 ± 0.01^bc^	1.03 ± 0.01^bc^	1.05 ± 0.04^bc^	1.21 ± 0.02^a^	1.04 ± 0.03^bc^
*C18:0*	6.10 ± 0.07^c^	5.97 ± 0.01^cd^	5.89 ± 0.02^de^	6.27 ± 0.02^b^	4.98 ± 0.01^g^	5.47 ± 0.01^f^	5.47 ± 0.08^f^	5.85 ± 0.06^de^	5.42 ± 0.02^f^	5.41 ± 0.03^f^	7.58 ± 0.02^a^	5.78 ± 0.01^e^
*C20:0*	1.37 ± 0.02^d^	1.58 ± 0.03^c^	1.40 ± 0.01^d^	1.52 ± 0.01^c^	1.36 ± 0.06^d^	2.10 ± 0.02^a^	1.43 ± 0.01^d^	1.23 ± 0.01^e^	2.06 ± 0.01^a^	1.91 ± 0.01^b^	0.78 ± 0.05^f^	1.52 ± 0.01^c^
*C21:0*	0.24 ± 0.05^d^	0.36 ± 0.02^bc^	0.45 ± 0.01^a^	0.48 ± 0.04^a^	0.19 ± 0.02^e^	0.18 ± 0.01^e^	0.31 ± 0.02^bc^	0.37 ± 0.01^b^	0.29 ± 0.01^c^	0.30 ± 0.01^c^	0.36 ± 0.01^bc^	0.26 ± 0.01^cd^
*C22:0*	0.98 ± 0.01^a^	0.64 ± 0.01^bc^	0.57 ± 0.01^bc^	0.62 ± 0.01^bc^	0.47 ± 0.20^c^	0.50 ± 0.01^bc^	0.55 ± 0.02^bc^	0.57 ± 0.01^bc^	0.60 ± 0.01^bc^	0.63 ± 0.02^bc^	0.70 ± 0.06^b^	0.60 ± 0.01^bc^
*C23:0*	0.18 ± 0.05^b^	0.23 ± 0.02^ab^	0.19 ± 0.04^b^	0.26 ± 0.01^a^	0.08 ± 0.01^c^	0.07 ± 0.03^c^	0.07 ± 0.01^c^	0.08 ± 0.01^c^	0.11 ± 0.01^c^	0.11 ± 0.01^c^	0.10 ± 0.01^c^	0.10 ± 0.01^c^
*C24:0*	0.32 ± 0.01^a^	0.23 ± 0.02^bc^	0.22 ± 0.02^bc^	0.23 ± 0.03^bc^	0.19 ± 0.01^c^	0.25 ± 0.01^b^	0.19 ± 0.01^c^	0.18 ± 0.02^c^	0.26 ± 0.02^b^	0.23 ± 0.01^bc^	0.22 ± 0.02^bc^	0.26 ± 0.01^b^
**ΣSFA**	**34.70 ± 0.04** ^**c**^	**36.81 ± 0.18** ^**ab**^	**36.00 ± 0.10** ^**b**^	**36.85 ± 0.13** ^**ab**^	**36.33 ± 1.62** ^**ab**^	**34.15 ± 0.21** ^**c**^	**36.68 ± 0.33** ^**ab**^	**36.89 ± 0.35** ^**ab**^	**34.34 ± 0.02** ^**c**^	**34.32 ± 0.25** ^**c**^	**38.35 ± 0.13** ^**a**^	**34.66 ± 0.13** ^**c**^
*C16:1*	5.07 ± 0.01^g^	6.20 ± 0.06^c^	6.49 ± 0.01^b^	6.07 ± 0.03^cd^	5.62 ± 0.18^f^	5.40 ± 0.06^f^	7.14 ± 0.01^a^	6.50 ± 0.01^b^	6.23 ± 0.03^c^	5.81 ± 0.01^e^	4.13 ± 0.04^h^	5.90 ± 0.03^de^
*C17:1*	0.31 ± 0.02^bc^	0.41 ± 0.01^a^	0.41 ± 0.04^a^	0.37 ± 0.02^ab^	0.35 ± 0.01^bc^	0.43 ± 0.03^a^	0.37 ± 0.01^ab^	0.37 ± 0.02^ab^	0.34 ± 0.02^bc^	0.33 ± 0.02^bc^	0.29 ± 0.01^c^	0.34 ± 0.03^bc^
*C18:1n9t*	0.11 ± 0.01^c^	0.15 ± 0.01^ab^	0.17 ± 0.02^ab^	0.16 ± 0.01^ab^	0.10 ± 0.01^c^	0.14 ± 0.01^bc^	0.15 ± 0.01^ab^	0.15 ± 0.01^ab^	0.14 ± 0.01^ab^	0.13 ± 0.01^bc^	0.19 ± 0.03^a^	0.12 ± 0.02^bc^
*C18:1n9c*	16.76 ± 0.06^e^	19.51 ± 0.08^c^	20.85 ± 0.11^a^	18.82 ± 0.04^d^	16.76 ± 0.15^e^	16.58 ± 0.01^e^	20.27 ± 0.31^b^	19.55 ± 0.16^c^	15.1 ± 0.02^f^	14.71 ± 0.11^f^	13.51 ± 0.0^6g^	17.06 ± 0.08^e^
*C20:1n9*	1.70 ± 0.18^b^	0.98 ± 0.01^d^	0.92 ± 0.19^d^	1.32 ± 0.02^c^	0.85 ± 0.02^d^	3.45 ± 0.02^a^	0.76 ± 0.04^d^	0.70 ± 0.01^d^	0.82 ± 0.01^d^	0.87 ± 0.03^d^	0.79 ± 0.03^d^	1.37 ± 0.03^c^
*C22:1n9*	0.20 ± 0.01^cd^	0.29 ± 0.01^bc^	0.31 ± 0.01^b^	0.22 ± 0.03^cd^	1.02 ± 0.02^a^	0.16 ± 0.01^e^	0.23 ± 0.01^cd^	0.15 ± 0.01^e^	0.19 ± 0.02^de^	0.21 ± 0.01^cd^	0.24 ± 0.04^cd^	0.18 ± 0.01^de^
*C24:1*	2.15 ± 0.10^a^	0.97 ± 0.06^ef^	0.88 ± 0.05^f^	0.92 ± 0.01^ef^	1.18 ± 0.08^d^	1.41 ± 0.04^c^	0.85 ± 0.05^f^	0.87 ± 0.01^f^	1.22 ± 0.07^d^	1.32 ± 0.01^cd^	1.04 ± 0.04^de^	1.60 ± 0.02^b^
**ΣMUFA**	**26.30 ± 0.22** ^**ab**^	**28.5 ± 0.05** ^**ab**^	**30.01 ± 0.07** ^**a**^	**24.85 ± 4.29** ^**bc**^	**25.86 ± 0.04** ^**bc**^	**27.55 ± 0.01** ^**ab**^	**29.76 ± 0.27** ^**ab**^	**28.28 ± 0.11** ^**ab**^	**24.03 ± 0.04** ^**bc**^	**23.37 ± 0.12** ^**cd**^	**20.18 ± 0.06** ^**d**^	**26.56 ± 0.05** ^**bc**^
*C18:2n6c*	2.14 ± 0.10^c^	2.36 ± 0.09^ab^	2.20 ± 0.04^bc^	2.30 ± 0.02^ab^	2.42 ± 0.06^a^	2.07 ± 0.03^c^	2.20 ± 0.12^bc^	2.32 ± 0.01^ab^	2.37 ± 0.13^ab^	2.43 ± 0.11^a^	2.38 ± 0.02^ab^	2.17 ± 0.07^cd^
*C18:3n6*	0.13 ± 0.01^b^	0.15 ± 0.02^ab^	0.16 ± 0.01^ab^	0.16 ± 0.01^ab^	0.17 ± 0.01^a^	0.13 ± 0.02^b^	0.17 ± 0.01^a^	0.16 ± 0.02^ab^	0.16 ± 0.01^ab^	0.15 ± 0.01^ab^	0.16 ± 0.01^ab^	0.13 ± 0.02^b^
*C18:3n3*	0.72 ± 0.02^f^	1.33 ± 0.06^cd^	1.35 ± 0.02^cd^	1.51 ± 0.03^b^	1.45 ± 0.06^bc^	1.10 ± 0.04^e^	1.26 ± 0.08^d^	1.39 ± 0.07^cd^	1.25 ± 0.04^d^	1.34 ± 0.02^cd^	1.80 ± 0.02^a^	1.08 ± 0.01^e^
*C20:2n6*	0.21 ± 0.01^h^	1.35 ± 0.01^de^	1.43 ± 0.02^cd^	1.50 ± 0.01^c^	1.94 ± 0.07^b^	1.23 ± 0.01^fg^	1.45 ± 0.01^c^	1.52 ± 0.02^c^	1.26 ± 0.01^ef^	1.34 ± 0.01^de^	2.52 ± 0.04^a^	1.16 ± 0.01^g^
*C20:3n6*	0.10 ± 0.01^a^	0.08 ± 0.01^a^	0.09 ± 0.01^a^	0.09 ± 0.01^a^	0.08 ± 0.03^a^	0.09 ± 0.01^a^	0.09 ± 0.00^a^	0.10 ± 0.01^a^	0.11 ± 0.00^a^	0.10 ± 0.01^a^	0.09 ± 0.01^a^	0.08 ± 0.01^a^
*C20:3n3*	0.07 ± 0.01^de^	0.19 ± 0.03^ab^	0.20 ± 0.01^ab^	0.23 ± 0.02^a^	0.11 ± 0.01^c^	0.05 ± 0.01^e^	0.09 ± 0.02^cd^	0.10 ± 0.00^cd^	0.11 ± 0.01^c^	0.12 ± 0.01^c^	0.16 ± 0.02^b^	0.07 ± 0.02^de^
*C20:4n6*	0.81 ± 0.01^bc^	0.78 ± 0.06^bc^	0.81 ± 0.02^bc^	0.82 ± 0.03^bc^	0.94 ± 0.01^b^	0.95 ± 0.20^b^	0.68 ± 0.01^c^	0.73 ± 0.04^bc^	1.22 ± 0.05^a^	1.28 ± 0.04^a^	1.20 ± 0.01^a^	0.86 ± 0.01^bc^
*C22:2n6*	0.37 ± 0.01^c^	0.40 ± 0.03^bc^	0.43 ± 0.03^b^	0.44 ± 0.04^b^	0.39 ± 0.01^bc^	0.41 ± 0.02^bc^	0.40 ± 0.03^bc^	0.42 ± 0.03^bc^	0.43 ± 0.01^b^	0.41 ± 0.01^bc^	0.50 ± 0.02^a^	0.38 ± 0.01^bc^
*C20:5n3*	6.47 ± 0.14^e^	7.10 ± 0.01^cd^	6.71 ± 0.05^de^	6.85 ± 0.03^de^	7.76 ± 0.44^b^	7.31 ± 0.04^bc^	7.05 ± 0.03^cd^	7.21 ± 0.04^cd^	8.95 ± 0.01^a^	8.81 ± 0.07^a^	7.05 ± 0.04^cd^	7.15 ± 0.04^cd^
*C22:6n3*	19.86 ± 0.04^a^	14.56 ± 0.01^fg^	13.78 ± 0.11^h^	14.25 ± 0.13^gh^	16.34 ± 0.21^e^	19.13 ± 0.08^b^	14.45 ± 0.06^fg^	14.86 ± 0.23^f^	19.03 ± 0.05^b^	19.35 ± 0.24^ab^	17.87 ± 0.06^d^	18.44 ± 0.07^c^
**ΣPUFA**	**30.86 ± 0.02** ^**d**^	**28.29 ± 0.08** ^**ef**^	**27.14 ± 0.24** ^**fg**^	**28.13 ± 0.29** ^**ef**^	**31.57 ± 0.85** ^**cd**^	**32.46 ± 0.12** ^**c**^	**27.82 ± 0.11** ^**ef**^	**28.79 ± 0.30** ^**e**^	**34.87 ± 0.03** ^**ab**^	**35.30 ± 0.30** ^**a**^	**33.70 ± 0.02** ^**b**^	**31.50 ± 0.12** ^**cd**^
∑PUFA/∑SFA	0.87 ± 0.06^b^	0.95 ± 0.01^ab^	0.76 ± 0.01^c^	0.78 ± 0.02^c^	1.02 ± 0.01^a^	1.03 ± 0.02^a^	0.88 ± 0.02^b^	0.91 ± 0.01^b^	0.89 ± 0.01^b^	0.77 ± 0.02^c^	0.75 ± 0.01^c^	0.76 ± 0.01^c^
∑PUFA/∑MUFA	1.22 ± 0.03^bc^	1.18 ± 0.02^cd^	0.93 ± 0.01^de^	1.02 ± 0.01^de^	1.45 ± 0.02^ab^	1.51 ± 0.02^a^	1.67 ± 0.02^a^	1.19 ± 0.01^c^	1.17 ± 0.01^cd^	0.99 ± 0.01^de^	0.90 ± 0.01^e^	1.15 ± 0.21^cd^
EPA + DHA	24.1 ± 0.64^d^	26.44 ± 0.12^b^	21.5 ± 0.09^ef^	22.06 ± 0.27^e^	27.97 ± 0.04^a^	28.16 ± 0.31^a^	24.91 ± 0.03^cd^	25.59 ± 0.11^bc^	26.33 ± 0.04^b^	21.66 ± 0.01^ef^	20.49 ± 0.16^g^	21.10 ± 0.16^fg^
∑n3	25.65 ± 0.71^c^	27.59 ± 0.12^b^	22.84 ± 0.08^de^	23.55 ± 0.28^de^	29.33 ± 0.02^a^	29.61 ± 0.33^a^	26.87 ± 0.05^b^	26.74 ± 0.12^b^	27.11 ± 0.03^b^	23.18 ± 0.04^d^	22.03 ± 0.18^e^	22.83 ± 0.18^de^
∑n6	5.93 ± 0.13^b^	4.87 ± 0.24^e^	4.98 ± 0.03^e^	5.24 ± 0.03^cd^	5.54 ± 0.03^cd^	5.69 ± 0.03^bc^	6.83 ± 0.03^a^	4.76 ± 0.04^e^	3.75 ± 0.05^f^	5.11 ± 0.04^de^	5.11 ± 0.06^de^	5.30 ± 0.11^cd^
∑n3/∑n6	4.33 ± 0.02^de^	5.67 ± 0.30^b^	4.59 ± 0.01^de^	4.49 ± 0.03^de^	5.29 ± 0.03^bc^	5.20 ± 0.08^bc^	3.93 ± 0.02^e^	5.62 ± 0.03^b^	7.24 ± 0.10^a^	4.53 ± 0.03^d^	4.31 ± 0.01^de^	4.31 ± 0.05^de^
∑n6/∑36	0.23 ± 0.01^ab^	0.18 ± 0.01^d^	0.22 ± 0.01^bc^	0.22 ± 0.02^b^	0.19 ± 0.01^d^	0.19 ± 0.01^cd^	0.25 ± 0.02^a^	0.18 ± 0.01^d^	0.14 ± 0.00^e^	0.22 ± 0.01^bc^	0.23 ± 0.00^ab^	0.23 ± 0.01^ab^
AI	0.79 ± 0.06^ab^	0.69 ± 0.01^c^	0.80 ± 0.01^ab^	0.79 ± 0.01^ab^	0.72 ± 0.02^bc^	0.72 ± 0.01^bc^	0.80 ± 0.01^ab^	0.72 ± 0.02^bc^	0.69 ± 0.02^c^	0.77 ± 0.02^ab^	0.75 ± 0.01^ab^	0.81 ± 0.06^a^
TI	0.32 ± 0.02^bc^	0.28 ± 0.02^de^	0.35 ± 0.01^a^	0.35 ± 0.01^a^	0.27 ± 0.02^e^	0.27 ± 0.02^e^	0.35 ± 0.02^a^	0.29 ± 0.00^cd^	0.29 ± 0.01^de^	0.35 ± 0.00^ab^	0.35 ± 0.01^a^	0.36 ± 0.02^a^
FLQ	25.7 ± 0.89^c^	28.08 ± 0.10^b^	22.81 ± 0.09^d^	23.48 ± 0.32^d^	30.0 ± 0.06^a^	30.29 ± 0.36^a^	27.01 ± 0.05^bc^	27.6 ± 0.14^b^	28.66 ± 0.01^ab^	23.14 ± 0.07^d^	21.99 ± 0.12^d^	23.52 ± 1.26^d^
h/H	1.86 ± 0.13^cd^	2.07 ± 0.02^ab^	1.83 ± 0.03^d^	1.85 ± 0.03^cd^	2.14 ± 0.03^a^	2.13 ± 0.02^a^	1.78 ± 0.01^d^	2.01 ± 0.01^abc^	2.02 ± 0.01^abc^	1.87 ± 0.01^cd^	1.91 ± 0.02^bcd^	1.86 ± 0.03^cd^
PI	1.24 ± 0.09^c^	1.49 ± 0.04^b^	1.09 ± 0.01^de^	1.12 ± 0.03^de^	1.65 ± 0.02^a^	1.63 ± 0.04^a^	1.19 ± 0.01^cd^	1.4 ± 0.02^b^	1.45 ± 0.01^b^	1.11 ± 0.01^de^	1.07 ± 0.02^e^	1.10 ± 0.02^de^

Different superscript letters denote significant statistical differences in the monthly data within the same column (*p* < 0.05). MUFA, monounsaturated fatty acids; PUFA: polyunsaturated fatty acids; SFA, saturated fatty acids; Σn–6 PUFA, total n–6 polyunsaturated fatty acid; Σn–3 PUFA, total n–3 polyunsaturated fatty acid; AI, Atherogenicity index; TI, thrombogenicity index; FLQ, fish lipid quality index; h/H, hypocholesterolemic/hypercholesterolemic ratio; PI, polyene index; n-3/n-6 denotes the ratio between total amount of n-3 and n-6 fatty acids.

**Table 4:** Seasonal fatty acid composition and nutritional quality indexes of anchovies from the southeastern Black Sea (%).

FA type	Autumn	Winter	Spring	Summer
*C4:0*	-	-	-	-
*C12:0*	0.05 ± 0.01^a^	0.06 ± 0.00^a^	0.07 ± 0.00^a^	0.05 ± 0.01^a^
*C13:0*	0.04 ± 0.01^a^	0.04 ± 0.01^a^	0.04 ± 0.00^a^	0.04 ± 0.00^a^
*C14:0*	5.76 ± 0.34^a^	6.08 ± 0.32^a^	6.40 ± 0.09^a^	5.86 ± 0.32^a^
*C15:0*	1.01 ± 0.10^a^	1.06 ± 0.16^a^	1.02 ± 0.09^a^	0.93 ± 0.03^a^
*C16:0*	18.92 ± 1.87^a^	18.84 ± 0.96^a^	18.80 ± 1.58^a^	18.82 ± 1.87^a^
*C17:0*	1.10 ± 0.01^a^	1.05 ± 0.08^a^	1.03 ± 0.02^a^	1.10 ± 0.09^a^
*C18:0*	5.98 ± 0.10^a^	5.57 ± 0.65^a^	5.58 ± 0.23^a^	6.25 ± 1.16^a^
*C20:0*	1.45 ± 0.11^a^	1.66 ± 0.38^a^	1.57 ± 0.43^a^	1.40 ± 0.57^a^
*C21:0*	0.35 ± 0.10^a^	0.28 ± 0.17^a^	0.32 ± 0.04^a^	0.30 ± 0.05^a^
*C22:0*	0.73 ± 0.21^a^	0.53 ± 0.07^a^	0.57 ± 0.02^a^	0.64 ± 0.05^a^
*C23:0*	0.20 ± 0.02^a^	0.13 ± 0.10^a^	0.08 ± 0.02^a^	0.10 ± 0.00^a^
*C24:0*	0.25 ± 0.05^a^	0.22 ± 0.03^a^	0.21 ± 0.04^a^	0.23 ± 0.02^a^
**ΣSFA**	**35.83 ± 1.06** ^**a**^	**35.77 ± 1.43** ^**a**^	**35.97 ± 1.41** ^**a**^	**35.77 ± 2.23** ^**a**^
*C16:1*	5.92 ± 0.75^a^	5.69 ± 0.37^a^	6.62 ± 0.46^a^	5.28 ± 0.99^a^
*C17:1*	0.37 ± 0.05^a^	0.38 ± 0.04^a^	0.36 ± 0.01^a^	0.32 ± 0.02^a^
*C18:1n9t*	0.14 ± 0.03^a^	0.13 ± 0.03^a^	0.14 ± 0.00^a^	0.14 ± 0.03^a^
*C18:1n9c*	19.04 ± 2.08^a^	17.38 ± 1.24^a^	18.30 ± 2.80^a^	15.09 ± 1.80^a^
*C20:1n9*	1.20 ± 0.43^a^	1.87 ± 1.38^a^	0.76 ± 0.06^a^	1.01 ± 0.31^a^
*C22:1n9*	0.26 ± 0.05^a^	0.46 ± 0.48^a^	0.19 ± 0.04^a^	0.21 ± 0.03^a^
*C24:1*	1.33 ± 0.70^a^	1.17 ± 0.24^a^	0.98 ± 0.20^a^	1.32 ± 0.28^a^
**ΣMUFA**	**28.27 ± 1.86** ^**a**^	**26.08 ± 1.36** ^**a**^	**27.35 ± 2.97** ^**a**^	**23.37 ± 3.19** ^**a**^
*C18:2n6c*	2.23 ± 0.11^a^	2.26 ± 0.17^a^	2.29 ± 0.08^a^	2.32 ± 0.13^a^
*C18:3n6*	0.14 ± 0.01^a^	0.15 ± 0.02^a^	0.16 ± 0.00^a^	0.14 ± 0.01^a^
*C18:3n3*	1.13 ± 0.35^a^	1.35 ± 0.22^a^	1.30 ± 0.07^a^	1.43 ± 0.33^a^
*C20:2n6*	0.99 ± 0.68^a^	1.55 ± 0.35^a^	1.41 ± 0.13^a^	1.67 ± 0.73^a^
*C20:3n6*	0.09 ± 0.01^a^	0.08 ± 0.00^a^	0.10 ± 0.00^a^	0.09 ± 0.01^a^
*C20:3n3*	0.15 ± 0.07^a^	0.13 ± 0.09^a^	0.10 ± 0.01^a^	0.11 ± 0.04^a^
*C20:4n6*	0.80 ± 0.01^a^	0.90 ± 0.07^a^	0.87 ± 0.29^a^	1.11 ± 0.22^a^
*C22:2n6*	0.40 ± 0.03^a^	0.41 ± 0.02^a^	0.41 ± 0.01^a^	0.43 ± 0.06^a^
*C20:5n3*	6.76 ± 0.31^a^	7.30 ± 0.45^a^	7.73 ± 1.05^a^	7.67 ± 0.98^a^
*C22:6n3*	16.06 ± 3.30^a^	16.57 ± 2.44^a^	16.11 ± 2.53^a^	18.55 ± 0.74^a^
**ΣPUFA**	**28.76 ± 1.90** ^**a**^	**30.72 ± 2.28** ^**a**^	**30.49 ± 3.82** ^**a**^	**33.50 ± 1.90** ^**a**^
∑PUFA/∑SFA	0.86 ± 0.09^a^	0.94 ± 0.14^a^	0.89 ± 0.01^a^	0.76 ± 0.01^a^
∑PUFA/∑MUFA	1.11 ± 0.15^a^	1.32 ± 0.26^a^	1.34 ± 0.28^a^	1.01 ± 0.12^a^
EPA + DHA	24.1 ± 2.47^a^	26.06 ± 3.46^a^	25.61 ± 0.71^a^	21.08 ± 0.58^a^
∑n3	25.36 ± 2.38^a^	27.49 ± 3.42^a^	26.90 ± 0.18^a^	22.68 ± 0.58^a^
∑n6	5.26 ± 0.58^a^	5.49 ± 0.22^a^	5.11 ± 1.57^a^	5.17 ± 0.10^a^
∑n3/∑n6	4.86 ± 0.71^a^	4.99 ± 0.43^a^	5.59 ± 1.65^a^	4.38 ± 0.12^a^
∑n6/∑n3	0.21 ± 0.02^a^	0.20 ± 0.01^a^	0.19 ± 0.05^a^	0.22 ± 0.00^a^
AI	0.76 ± 0.06^a^	0.74 ± 0.04^a^	0.73 ± 0.05^a^	0.77 ± 0.03^a^
TI	0.31 ± 0.03^a^	0.29 ± 0.04^a^	0.31 ± 0.03^a^	0.35 ± 0.00^a^
FLQ	25.53 ± 2.63	27.92 ± 3.85	27.75 ± 0.83	22.88 ± 0.79
h/H	1.92 ± 0.13^a^	2.04 ± 0.16^a^	1.93 ± 0.13^a^	1.88 ± 0.02^a^
PI	1.27 ± 0.20^a^	1.46 ± 0.30^a^	1.34 ± 0.13^a^	1.09 ± 0.02^a^

Different superscript letters denote significant statistical differences in the seasonal data within the same column (*p* < 0.05). MUFA: monounsaturated fatty acids; PUFA: polyunsaturated fatty acids; SFA, saturated fatty acids; Σn–6 PUFA, total n–6 polyunsaturated fatty acid; Σn–3 PUFA, total n–3 polyunsaturated fatty acid; AI, Atherogenicity index; TI, Thrombogenicity index; FLQ, fish lipid quality index; h/H, hypocholesterolemic/hypercholesterolemic ratio; PI, polyene index; n-3/n-6 denotes the ratio between total amount of n-3 and n-6 fatty acids.

#### 3.2.1 Saturated fatty acids (SFAs)

The muscle fillet had the highest total saturated fatty acid (∑SFA) content in July (38.35%) and the lowest in February (34.15%), with statistically significant differences observed across the months (*p* < 0.05). Seasonally, ∑SFA values displayed similar trends, though the differences between seasons were not statistically significant (*p* > 0.05). Previous studies have reported ∑SFA levels in anchovy muscle tissue ranging from 31.6 to 38.2% (8, 21, 32–34), which align with the findings of the present study. Palmitic acid (C16:0) was identified as the dominant saturated fatty acid, ranging from 16.97 to 20.89%. Interestingly, C16:0 levels were relatively lower in May and June, coinciding with the spawning period. This fatty acid was also found in lower amounts during the spring and early summer seasons. Additionally, stearic acid (C18:0, 5.57–6.25%) and myristic acid (C14:0, 5.76–6.40%) were also major SFAs, serving as key energy sources for metabolism and growth. Monthly variations in these fatty acids were statistically significant (*p* < 0.05), though seasonal differences were not significant (*p* > 0.05). These results were consistent with previous findings (8, 22).

#### 3.2.2 Monounsaturated fatty acids (MUFAs)

Total monounsaturated fatty acids (∑MUFA) were lowest in July (20.18%) and highest in November (30.01%), with significant monthly variation (*p* < 0.05). Seasonally, ∑MUFA values were lowest in the summer (23.37%) and highest in the autumn (28.27%), though seasonal differences were not significant (*p* > 0.05). These results were consistent with earlier studies reported by Öksüz and Özyılmaz (35), Öksüz et al. (36), Turhan et al. (34), and Tufan et al. (23). Oleic acid (C18:1n9c) was the predominant MUFA, ranging from 13.51–20.85%, with significant monthly differences (*p* < 0.05). Seasonally, its lowest level was in summer (15.09%) and highest in autumn (19.04%), though differences were not significant (*p* > 0.05). Literature values for C18:1n9c ranged from 14.67–20.26% (35) and 11.31–16.15% (23), consistent with the present study (Table 4).

**Table 6:** Monthly amino acid composition of anchovies from the southeastern Black Sea (mg/100 g).

Amino acid	2021				2022							
	September	October	November	December	January	February	March	April	May	June	July	August
Histidine*	757 ± 10^e^	1,127 ± 34^abc^	1,025 ± 16^bcd^	993 ± 2^bcd^	1,205 ± 22^a^	923 ± 74^de^	1,171 ± 53^ab^	1,300 ± 83^a^	986 ± 73^cd^	1,287 ± 16^a^	888 ± 39^de^	1,151 ± 4^abc^
Threonine*	659 ± 5^a^	683 ± 108^a^	760 ± 95^a^	652 ± 24^a^	665 ± 41^a^	849 ± 15^a^	734 ± 25^a^	737 ± 66^a^	700 ± 95^a^	737 ± 4^a^	814 ± 16^a^	778 ± 73^a^
Valine*	849 ± 4^abc^	920 ± 6^a^	877 ± 2^ab^	859 ± 6^abc^	932 ± 18^a^	811 ± 36^bc^	932 ± 2^a^	811 ± 1^bc^	802 ± 26^bc^	775 ± 12^c^	877 ± 32^ab^	875 ± 44^ab^
Methionine*	496 ± 4^a^	373 ± 34^a^	480 ± 24^a^	480 ± 1^a^	542 ± 39^a^	511 ± 44^a^	446 ± 8^a^	459 ± 10^a^	462 ± 49^a^	465 ± 9^a^	384 ± 166^a^	507 ± 26^a^
Isoleucine*	811 ± 2^cde^	893 ± 11^ab^	859 ± 8^bcd^	850 ± 12^bcd^	920 ± 5^a^	809 ± 20^cde^	878 ± 1^ab^	788 ± 9^e^	807 ± 24^de^	777 ± 12^e^	852 ± 27^bcd^	862 ± 12^bc^
Leucine*	1,315 ± 2^bc^	1,442 ± 59^ab^	1,425 ± 39^ab^	1,385 ± 4^abc^	1,508 ± 28^a^	1,349 ± 38^bc^	1,494 ± 4^a^	1,281 ± 13^c^	1,387 ± 50^abc^	1,318 ± 13^bc^	1,406 ± 22^abc^	1,417 ± 42^ab^
Phenylalanine*	742 ± 2^ab^	770 ± 21^ab^	764 ± 29^ab^	773 ± 2^ab^	815 ± 16^a^	705 ± 49^b^	782 ± 3^ab^	696 ± 49^b^	689 ± 44^b^	691 ± 10^b^	696 ± 6^b^	741 ± 1^ab^
Lysine*	3,096 ± 25^ab^	3,112 ± 317^ab^	3,386 ± 78^ab^	3,489 ± 14^a^	3,450 ± 182^ab^	3,210 ± 88^ab^	2,874 ± 25^b^	2,919 ± 110^ab^	2,974 ± 215^ab^	3,020 ± 64^ab^	3,206 ± 78^ab^	3,346 ± 208^ab^
Aspartic acid	1,376 ± 9^a^	1,574 ± 135^a^	1,358 ± 207^a^	1,304 ± 5^a^	1,328 ± 277^a^	1,578 ± 119^a^	1,278 ± 16^a^	1,541 ± 90^a^	1,471 ± 102^a^	1,477 ± 27^a^	1,582 ± 15^a^	1,493 ± 131^a^
Glutamic acid	2,289 ± 5^a^	2,356 ± 136^a^	2,319 ± 64^a^	2,178 ± 14^a^	2,244 ± 279^a^	2,388 ± 97^a^	2,517 ± 7^a^	2,327 ± 7^a^	2,456 ± 114^a^	2,261 ± 29^a^	2,466 ± 51^a^	2,365 ± 58^a^
Serine	623 ± 4^ab^	599 ± 57^ab^	618 ± 12^ab^	600 ± 3^ab^	592 ± 38^ab^	663 ± 75^ab^	703 ± 9^a^	735 ± 9^a^	558 ± 29^b^	693 ± 2^ab^	669 ± 43^ab^	660 ± 45^ab^
Glycine	844 ± 1^a^	865 ± 90^a^	884 ± 54^a^	768 ± 1^a^	914 ± 88^a^	856 ± 2^a^	917 ± 25^a^	888 ± 32^a^	844 ± 44^a^	879 ± 7^a^	921 ± 20^a^	941 ± 41^a^
Arginine	837 ± 2^a^	733 ± 90^a^	846 ± 1^a^	883 ± 19^a^	848 ± 175^a^	746 ± 54^a^	945 ± 12^a^	925 ± 36^a^	853 ± 13^a^	704 ± 12^a^	878 ± 19^a^	875 ± 47^a^
Alanine	1,112 ± 3^b^	1,155 ± 38^b^	1,133 ± 21^b^	1,069 ± 3^b^	1,178 ± 7^ab^	1,120 ± 48^b^	1,278 ± 7^a^	1,123 ± 21^b^	1,087 ± 68^b^	1,061 ± 14^b^	1,103 ± 21^b^	1,138 ± 30^b^
Proline	626 ± 7^a^	646 ± 49^a^	648 ± 52^a^	589 ± 1^a^	703 ± 121^a^	640 ± 2^a^	674 ± 5^a^	659 ± 7^a^	616 ± 28^a^	624 ± 4^a^	679 ± 1^a^	709 ± 3^a^
Tyrosine	556 ± 4^c^	589 ± 25^abc^	611 ± 40^abc^	569 ± 2^abc^	642 ± 24^a^	582 ± 11^abc^	581 ± 4^abc^	563 ± 17^bc^	601 ± 16^abc^	545 ± 15^c^	581 ± 21^abc^	638 ± 13^ab^
∑EAA*	8,727 ± 37^d^	9,324 ± 439^abc^	9,579 ± 154^abc^	9,484 ± 11^abc^	10,039 ± 17^a^	9,170 ± 337^bcd^	9,313 ± 59^abc^	8,993 ± 49^bcd^	8,809 ± 42^cd^	9,074 ± 142^bcd^	9,125 ± 188^bcd^	9,681 ± 236^ab^
∑NEAA	8,265 ± 8^a^	8,519 ± 326^a^	8,419 ± 386^a^	7,961 ± 2^a^	8,453 ± 528^a^	8,576 ± 274^a^	8,894 ± 14^a^	8,764 ± 86^a^	8,489 ± 152^a^	8,245 ± 114^a^	8,882 ± 21^a^	8,822 ± 222^a^
∑EAA/∑NEAA	1.06 ± 0.01^ab^	1.10 ± 0.09^ab^	1.14 ± 0.03^ab^	1.19 ± 0.01^a^	1.19 ± 0.05^a^	1.07 ± 0.01^ab^	1.05 ± 0.01^b^	1.03 ± 0.01^b^	1.04 ± 0.02^b^	1.10 ± 0.01^ab^	1.03 ± 0.02^b^	1.10 ± 0.01^ab^

Different superscript letters denote significant statistical differences in the monthly data within the same column (*p* < 0.05). ∑EAA*, Total essential amino acids. ∑NEAA, Total non-essential amino acids.

#### 3.2.3 Polyunsaturated fatty acids (PUFAs)

Total polyunsaturated fatty acids (ΣPUFA) were lowest in November (27.14%) and highest in June (35.30%). Seasonally, ΣPUFA was lowest in autumn (28.76%) and highest in summer (33.50%). These levels are consistent with previous Black Sea anchovy studies (8, 23, 35). However, regional differences exist: non-spawning anchovies in the North Catalan Sea contained 39.34% ΣPUFA, while spawning individuals had 50.90% (37); anchovies from the Yellow Sea had 23.88% (54); Mediterranean anchovies showed 22.12–28.29% (38); and Argentine anchovies contained 44.92% (39). Such variability may result from species, geography, diet, season, and age.

**Table 7:** Seasonal amino acid composition of anchovies from the southeastern Black Sea (mg/100 g).

Amino acid	Autumn	Winter	Spring	Summer
Histidine*	969.67 ± 191.10^a^	1040.33 ± 146.83^a^	1152.33 ± 157.83^a^	1108.67 ± 202.84^a^
Threonine*	700.66 ± 52.76^a^	722.00 ± 110.17^a^	723.66 ± 20.55^a^	776.33 ± 38.52^a^
Valine*	882.00 ± 35.76^a^	867.33 ± 60.92^a^	848.33 ± 72.59^a^	842.33 ± 58.32^a^
Methionine*	449.66 ± 66.87^a^	511.00 ± 31.00^a^	455.66 ± 8.50^a^	452.00 ± 62.52^a^
Isoleucine*	854.33 ± 41.19^a^	859.66 ± 56.12^a^	824.33 ± 47.43^a^	830.33 ± 46.45^a^
Leucine*	1394.00 ± 68.94^a^	1414.00 ± 83.37^a^	1387.33 ± 106.50^a^	1380.33 ± 54.26^a^
Phenylalanine*	758.66 ± 14.74	764.33 ± 55.50	722.33 ± 51.79	709.33 ± 27.53
Lysine*	3198.00 ± 163.00^ab^	3383.00 ± 151.08^a^	2922.33 ± 50.08^b^	3190.67 ± 163.54^ab^
Aspartic acid	1436.00 ± 119.85^a^	1403.33 ± 151.74^a^	1430.00 ± 136.20^a^	1517.33 ± 56.57^a^
Glutamic acid	2321.33 ± 33.56^a^	2270.00 ± 107.38^a^	2433.33 ± 97.00^a^	2364.00 ± 102.50^a^
Serine	613.33 ± 12.66^a^	618.33 ± 38.88^a^	665.33 ± 94.32^a^	674.00 ± 17.05^a^
Glycine	864.33 ± 20.00^a^	846.00 ± 73.51^a^	883.00 ± 36.75^a^	913.66 ± 31.64^a^
Arginine	805.33 + 62.80^a^	825.66 ± 71.17^a^	907.66 ± 48.38^a^	819.00 ± 99.60^a^
Alanine	1133.33 ± 21.50^a^	1122.33 ± 54.53^a^	1162.67 ± 101.49^a^	1100.67 ± 38.55^a^
Proline	640.00 ± 12.16^a^	644.00 ± 57.10^a^	649.66 ± 30.10^a^	670.66 ± 43.10^a^
Tyrosine	585.33 ± 27.68^a^	597.66 ± 38.94^a^	581.66 ± 19.00^a^	588.00 ± 46.89^a^
∑EAA*	9210.08 ± 437.47^a^	9564.58 ± 439.95^a^	9038.67 ± 254.77^a^	9293.50 ± 336.57^a^
∑NEAA	8401.17 ± 127.69^a^	8330.42 ± 325.43^a^	8716.00 ± 206.72^a^	8649.83 ± 351.90^a^
∑EAA/∑NEAA	1.10 ± 0.04^a^	1.15 ± 0.07^a^	1.04 ± 0.01^a^	1.08 ± 0.04^a^

Different superscript letters denote significant statistical differences in the seasonal data within the same column (*p* < 0.05). ∑EAA*, total essential amino acids. ∑NEAA, total non-essential amino acids.

Docosahexaenoic acid (DHA; C22:6n3) and eicosapentaenoic acid (EPA; C20:5n3) are essential PUFAs that play a critical role in cellular function, neural development, and cardiovascular health. Throughout the year, DHA and EPA were the most abundant PUFAs. DHA levels were lowest in November at 13.78% and reached their highest rate in September at 19.86%, showing significant monthly variation (*p* < 0.05). However, DHA levels were relatively consistent across autumn, winter, and spring, peaking at 18.55% in summer. This study found that seasonal variations were not significantly different in DHA content (*p* > 0.05). The lowest EPA content was recorded in autumn at 6.76%, while the highest was observed in summer at 7.67%. However, the difference between the seasons was not statistically significant (*p* > 0.05). Nevertheless, the levels of EPA and DHA were higher during the anchovy spawning period, which occurs from May to September, compared to other months. Özsüz and Özyılmaz (35) reported that the DHA content in anchovies collected during the fishing season ranged from 14.03 to 19.04%. Similarly, Öksüz et al. (36) determined the DHA content of Black Sea anchovies to be 16.40%. Tufan et al. (23) found that the DHA content in anchovies collected during the fishing season in the Black Sea ranged from 10.4 to 22.34%. The findings of the present study align closely with those reported in the literature. Additionally, the observed levels of EPA were in line with earlier findings (8, 33).

#### 3.2.4 Fatty acids quality index parameters

The C18:2/C18:3 ratio remained well below the cardiovascular health threshold of 5 (20). The ∑PUFA/∑SFA ratio varied significantly across months and seasons (*p* < 0.05), ranging from 0.75–1.02 monthly, with the lowest in summer (0.76) and highest in winter (0.94). These findings suggest that environmental factors, particularly temperature fluctuations, may significantly influence the fatty acid metabolism of organisms. The lower ∑PUFA/∑SFA ratio observed during the summer months may be attributed to elevated ambient temperatures, which could alter energy metabolism and affect the composition of dietary inputs. From a nutritional standpoint, an increased ∑PUFA/∑SFA ratio is regarded as a key indicator of favorable lipid profiles that support cardiovascular health (40). Thus, the higher values observed in the winter months suggest a more beneficial fatty acid composition, supporting the notion that seasonal changes impact dietary lipid quality. In conclusion, the results highlight that seasonal fluctuations in the ∑PUFA/∑SFA ratio should be considered when evaluating both food quality and public health. The values observed in this study align with previous findings ((8, 23); Gençbay & Turhan (32)). In general, the ∑PUFA/∑SFA ratio in human diets is recommended to be above 0.45 to reduce the risk of cardiovascular diseases (CVD) and chronic diseases such as cancer. A ratio below 0.45 is considered undesirable, as it may contribute to increased blood cholesterol levels (55). Moreover, a ratio of ≥0.4 is recommended for better cardiovascular health (CVH) (41). The results of this study indicate that the ∑PUFA/∑SFA ratio exceeded this threshold across all months and seasons, demonstrating the high nutritional value of anchovies.

The ∑PUFA/∑MUFA ratio ranged from 0.90 (July) to 1.67 (March), with significant monthly variation (*p* < 0.05), but no seasonal significance (*p* > 0.05). Tufan et al. (23) reported ∑PUFA/∑MUFA ratios ranging from 1.96 to 1.49 in anchovies collected during the fishing season, which aligns with the findings of the present study.

**Figure 4:**
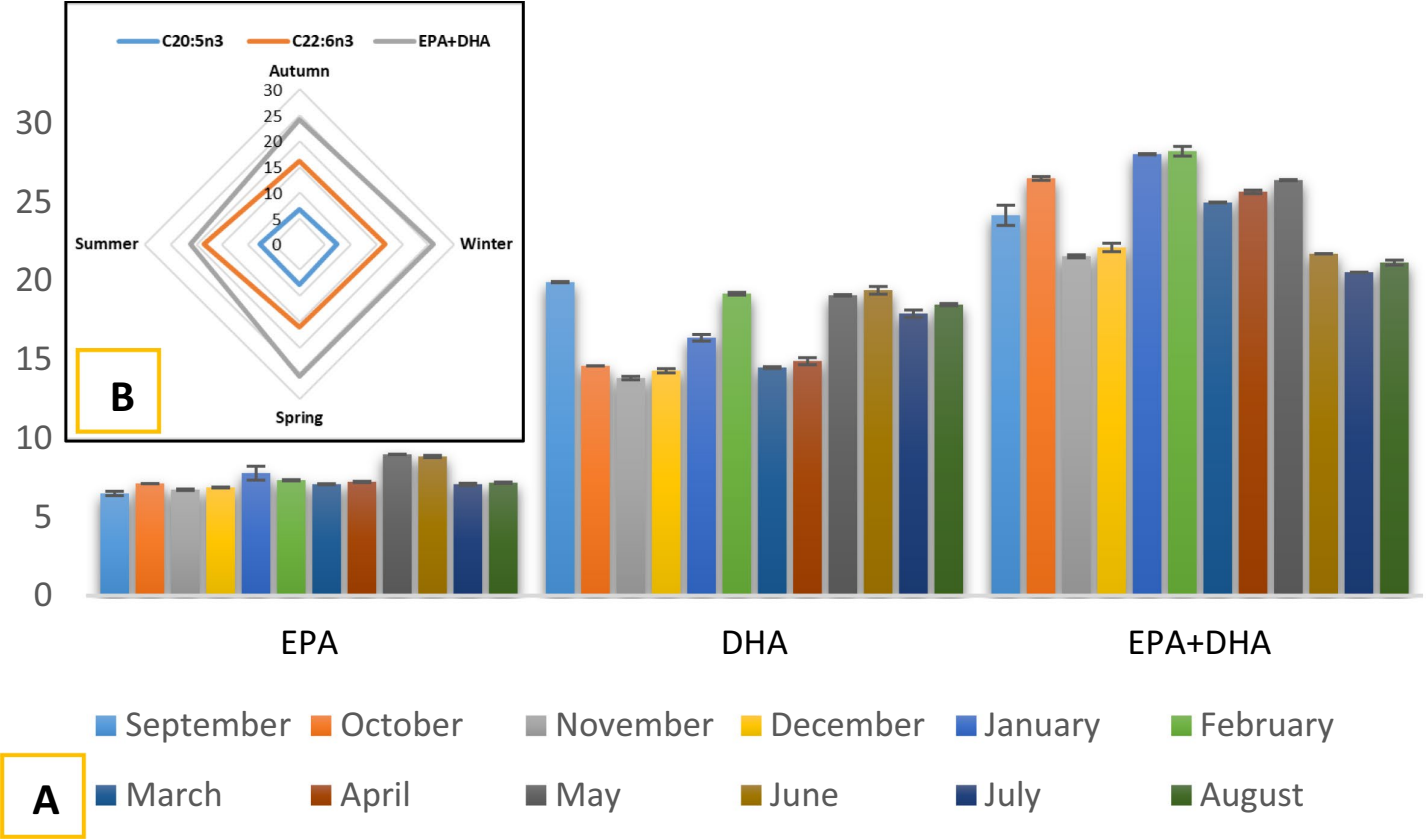
Monthly **(A)** and seasonal **(B)** changes in EPA, DHA, and EPA + DHA contents of anchovies from the Southeastern Black Sea.

The highest monthly EPA + DHA content occurred in January (27.97%) and February (28.16%), while the lowest was in July (20.49%) and August (21.10%), with significant monthly variation (*p* < 0.05). Seasonally, the EPA + DHA was highest in winter (26.06%) and lowest in summer (21.08%); however, differences were not significant (*p* > 0.05) (Figure 4). Previous studies, such as those by Tufan et al. (23), reported EPA + DHA levels ranging from 24.15 to 30.79% in anchovies collected during the fishing season. Similarly, Öğretmen (8) found EPA + DHA levels were between 30.7 and 27.2% in anchovies from various regions of the Black Sea. These findings align with the EPA + DHA levels observed in the current study. The Food and Agriculture Organization (42) recommends daily EPA + DHA intakes of 100–150 mg for children aged 2–4 years, 200–250 mg for children aged 6–10 years, and at least 300 mg for pregnant women (Table 5). Furthermore, The European Food Safety Authority (43) and the Turkish Ministry of Health (22) published similar guidelines, recommending a daily intake of EPA + DHA of 250 mg. Based on these recommendations, the observed EPA + DHA levels indicate that consuming ~20 g of anchovies per day can meet daily requirements (Table 5).

**Table 5:** The amount of anchovies that should be consumed seasonally to meet the recommended daily EPA + DHA requirement.

Seasons	Children aged 2–4	Children aged 6–10	Pregnant
EPA + DHA Recommendation (Ref: FAO, 2010)	100–150 mg/day	200-250 mg/day	At least 300 mg/day
Autumn	3.72–5.58 g	7.44–9.30 g	11.16 g
Winter	2.71–4.07 g	5.42–6.78 g	8.14 g
Spring	3.32–4.99 g	6.66–8.32 g	9.99 g
Summer	6.23–9.35 g	12.47–15.58 g	18.70 g

Monthly ∑n3 levels peaked in January and February, and the lowest were in July and August. Seasonally, ∑n3 was highest in winter (27.49%) and lowest in summer (22.68%). Significant variation was observed monthly (*p* < 0.05), but not seasonally (*p* > 0.05). Previous studies by Tufan et al. (23) reported ∑n3 amounts in *E. encrasicolus* ranging from 25.40 to 32.60%, while Sun et al. (33) detected 23.5% in *E. japonicus*, and Öğretmen (8) found 28.9 to 32.1% in *E. encrasicolus*. These findings are comparable to the results of the present study. For ∑n6, the lowest value was recorded in May (3.75%), while the highest was in January (6.83%), with significant monthly variation (*p* < 0.05). Seasonally, ∑n6 was lowest in spring (5.11%) and highest in winter (5.49%), with no significant differences observed between the seasons (*p* > 0.05). Czerner et al. (39) reported ∑n6 as being 8.69% in *E. anchoita* collected in Argentina, which is higher than the present study’s findings, likely due to species and geographical differences. Studies by Öğretmen (8), Öksüz and Özyılmaz (35), and Tufan et al. (23) found ∑n6 levels in *E. encrasicolus* from the Black Sea to be similar to the results of this study.

The ∑n3/∑n6 ratio is a key indicator of the nutritional value of fish oil. Therefore, it is essential to consume products that are rich in ∑n3 and low in ∑n6. Öğretmen (8) reported that foods with a high ∑n3/∑n6 ratio can effectively reduce the risk of coronary heart disease and cancer. In this study, the ∑n3/∑n6 ratio was lowest in March at 3.93% and highest in May at 7.24%. Seasonally, the lowest ratio of 4.38% was recorded in summer, while the highest ratio of 5.59% was observed in spring. Öğretmen (8) noted that the ∑n3/∑n6 ratio should be above 1. The findings of this study were well above this threshold, indicating that anchovy is a food with high nutritional value. Kocatepe et al. (19) reported the ∑n3/∑n6 ratios in anchovies from the Aegean Sea, Black Sea, and Mediterranean Sea to be 6.76, 2.37, and 1.29%, respectively. Öksüz and Özyılmaz (35) found the ratio ranged between 5.85 and 8.07% during the fishing season, while Tufan et al. (23) recorded ratios between 4.63 and 10.13%. Additionally, Öğretmen (8) observed that the ∑n3/∑n6 ratio for anchovies collected from different regions of the Black Sea ranged from 5.12 to 6.27%. A comparison of the ∑n3/∑n6 ratios from previous studies with the results of this study revealed both similarities and differences. These variations may be attributed to factors such as the nutritional status of the fish, gender, age, and fishing location. The quality of the fish meat is assessed based on the ∑n6/∑n3 ratio. A lower ratio of omega-6 to omega-3 fatty acids can help reduce cholesterol levels in the blood, thus preventing coronary heart disease. Conversely, a higher rate increases the risk of heart disease. The UK Department of Health recommends that the ∑n6/∑n3 ratio should be below 4 to lower the risk of heart disease (3). In the present study, the ∑n6/∑n3 ratio ranged from 0.14 to 0.25 in monthly samples and from 0.19 to 0.22 in seasonally collected samples, both of which are well below the recommended limit of 4 set by the UK Department of Health. This indicates that anchovies were a health-beneficial food suitable for year-round consumption. Furthermore, the ∑n6/∑n3 ratio observed in this study is consistent with findings from previous research (8, 23).

This study not only calculated the ratios of SFAs, MUFAs, PUFAs, and n-3 and n-6 PUFAs, but also assessed several key parameters related to intramuscular fat quality. These parameters include the Atherogenic Index (AI), Thrombotic Index (TI), Fatty Acid Quality (FLQ), h/H ratio, and Polyene Index (PI). For a healthy diet, it is recommended that the AI be less than 1 and the TI be below 0.5 (44). Low values for both indices indicate that the fatty acids have better nutritional quality and may lower the risk of coronary heart disease (45). In this study, the AI ranged between 0.69–0.80 (monthly, *p* < 0.05) and 0.73–0.77 (seasonal, *p* > 0.05), while the TI ranged from 0.27–0.36 (monthly) and 0.29–0.35 (seasonal). Both indices were below recommended thresholds, indicating anchovy consumption may reduce coronary heart disease risk.

The FLQ index is utilized to assess the quality of fish lipids by calculating the combined percentage of EPA + DHA in total fatty acids. Given that seafood is rich in both EPA + DHA, this index is particularly relevant for evaluating seafood (2, 46, 47). Additionally, high levels of FLQ are associated with a reduced risk of cardiovascular disease, hypertension, heart disease, and dementia (48). In this study, the highest FLQ values were recorded in January (30.0) and February (30.29), while the lowest value was observed in July (21.99). The variations in FLQ values among the anchovy samples collected each month were found to be statistically significant (*p* < 0.05). Seasonally, FLQ values ranged from 22.88 to 27.92. Öğretmen (8) reported FLQ values for anchovies collected from different regions of the Black Sea were between 29.3 and 32.7. Meanwhile, Kocatepe et al. (19) measured FLQ values for anchovies from the Aegean, Black Sea, and Mediterranean regions, reporting values of 39.37, 16.48, and 19.01, respectively.

The h/H ratio is an index that measures the relationship between unsaturated and saturated fatty acids, and it is linked to cholesterol metabolism. A higher h/H ratio suggests that the dietary lipid supply is of better quality (47). An elevated h/H ratio indicates greater nutritional value, which is believed to be more beneficial for human health (44, 45). In this study, the highest h/H indices were recorded in January (2.14) and February (2.13), while the lowest was observed in March (1.78). The differences in the monthly h/H indices of the anchovies collected were statistically significant (*p* < 0.05). When assessed by season, the h/H ratio was lowest in summer (1.88) and highest in winter (2.04), also with statistical significance (*p* < 0.05). Öğretmen (8) reported the h/H values for anchovies collected from different regions of the Black Sea to range between 1.83 and 2.40, which aligns with the findings of the current study.

PI index is a valuable measure for evaluating the quality of PUFAs and assessing any damage they may have incurred (47). A decrease in the PI index indicates a decline in PUFA quality (8). In the present study, the PI index ranged from 1.65 to 1.07 (monthly) and from 1.46 to 1.09 (seasonal). Significant monthly variation was observed (*p* < 0.05), while seasonal differences were not significant (*p* > 0.05). The PI values were consistent with those reported by Öğretmen (8).

### 3.3 Changes in total amino acid (TAA)

The monthly and seasonal variations in the total amino acid content of European anchovy fillets are detailed in Tables 6, 7. This study analyzed 16 amino acids, which included 8 essential amino acids (EAAs), 5 non-essential amino acids (NEAAs), and 3 semi-essential amino acids (SEAAs). Among the NEAAs, glutamic acid was found to be the most prevalent, consistent with previous research by Öğretmen (8) on *E. encrasicolus*, Sun et al. (33) on *E. japonicus*, Pariona-Velarde et al. (20) on *E. ringens*, and Gencbay and Turhan (32) on *E. encrasicolus*. The highest concentration of glutamic acid was recorded in March at 2517 mg/100 g, while the lowest concentration was in December at 2178 mg/100 g. Seasonal differences also emerged, with glutamic acid levels peaking in spring at 2433 mg/100 g and reaching their lowest in winter at 2270 mg/100 g. However, the variations between months and seasons were statistically insignificant (*p* > 0.05). These results align with those of Öğretmen (8), who reported glutamic acid concentrations ranging from 1,658 to 2,549 mg/100 g in anchovy fillets collected along the Black Sea coast. Following glutamic acid, the second and third most abundant NEAAs were aspartic acid and alanine, respectively. Alanine levels displayed seasonal fluctuations throughout the year, with significant monthly differences observed (*p* < 0.05). Conversely, aspartic acid levels did not show significant variations between months and seasons (*p* > 0.05). Monthly analysis revealed significant differences in the concentrations of tyrosine and serine (*p* < 0.05). Notably, tyrosine was found to be the least abundant NEAA, which is consistent with the findings of Gencbay and Turhan (32). The concentration of ∑NEAA in anchovy fillets ranged from 7,961 mg/100 g in December to 8,894 mg/100 g in March. There were no significant differences in the levels of ∑NEAA observed across the different months (*p* > 0.05). The highest levels of ∑NEAA were found in the spring and summer seasons; however, no significant seasonal differences were identified (*p* > 0.05).

The concentration of ∑EAA showed seasonal variations, with the lowest amount recorded in September at 8727 mg/100 g and the highest in January at 10039 mg/100 g. The levels of ∑EAA fluctuated throughout the year, with significant differences noted between the months (*p* < 0.05). These results align with findings from Öğretmen (8) but differ from those reported by Thanathornvarakul et al. (49) and Pariona-Velarde et al. (20). Such discrepancies may stem from factors like the species of anchovy, geographical location, and fishing times. Seasonally, the highest amount of ∑EAA was found in winter at 9564.58 mg/100 g, while the lowest was observed in spring at 9038.67 mg/100 g. The high levels of EAA in anchovies during the winter season are thought to be associated with metabolic changes due to decreasing water temperatures, the quality of dietary protein intake, and the onset of the reproductive period. However, the 525.91 mg/100 g difference between these seasons was not significant (*p* > 0.05). Lysine, an essential amino acid (EAA), is vital for optimal growth, and a deficiency in lysine can lead to immune system dysfunction. Additionally, lysine is commonly used to prevent cold sores and can be taken orally or applied directly to the skin (7). Among the EAAs, lysine was identified as the most predominant, aligning with the findings of Öğretmen (8), Pariona-Velarde et al. (20), and Gencbay and Turhan (32). The lysine content in anchovy fillets varied monthly, ranging from 2,974 to 3,346 mg/100 g, with significant differences noted between months (*p* < 0.05). Seasonally, the highest lysine concentration was recorded in winter at 3383 mg/100 g, while the lowest was observed in spring at 2922 mg/100 g. The difference in lysine levels across seasons was statistically significant (*p* < 0.05). Leucine, the second most abundant EAA after lysine, peaked in January at 1508 mg/100 g and in winter at 1414 mg/100 g. The lowest amounts were found in April at 1281 mg/100 g and during the spring at 1387 mg/100 g. While there were significant differences in leucine levels among the months (*p* < 0.05), seasonal variations were not significant (*p* > 0.05). According to the FAO/WHO/UNU (50) guidelines, the daily lysine and leucine requirements for children aged 10 to 12 years are 60 mg and 45 mg per kilogram of body weight, respectively. For a child weighing 30 kg, this translates to a daily requirement of 180 mg of lysine and 135 mg of leucine. Based on the current study’s findings, consuming 50 grams of anchovy fillet daily would meet the annual requirements for both leucine and lysine.

**Figure 5:**
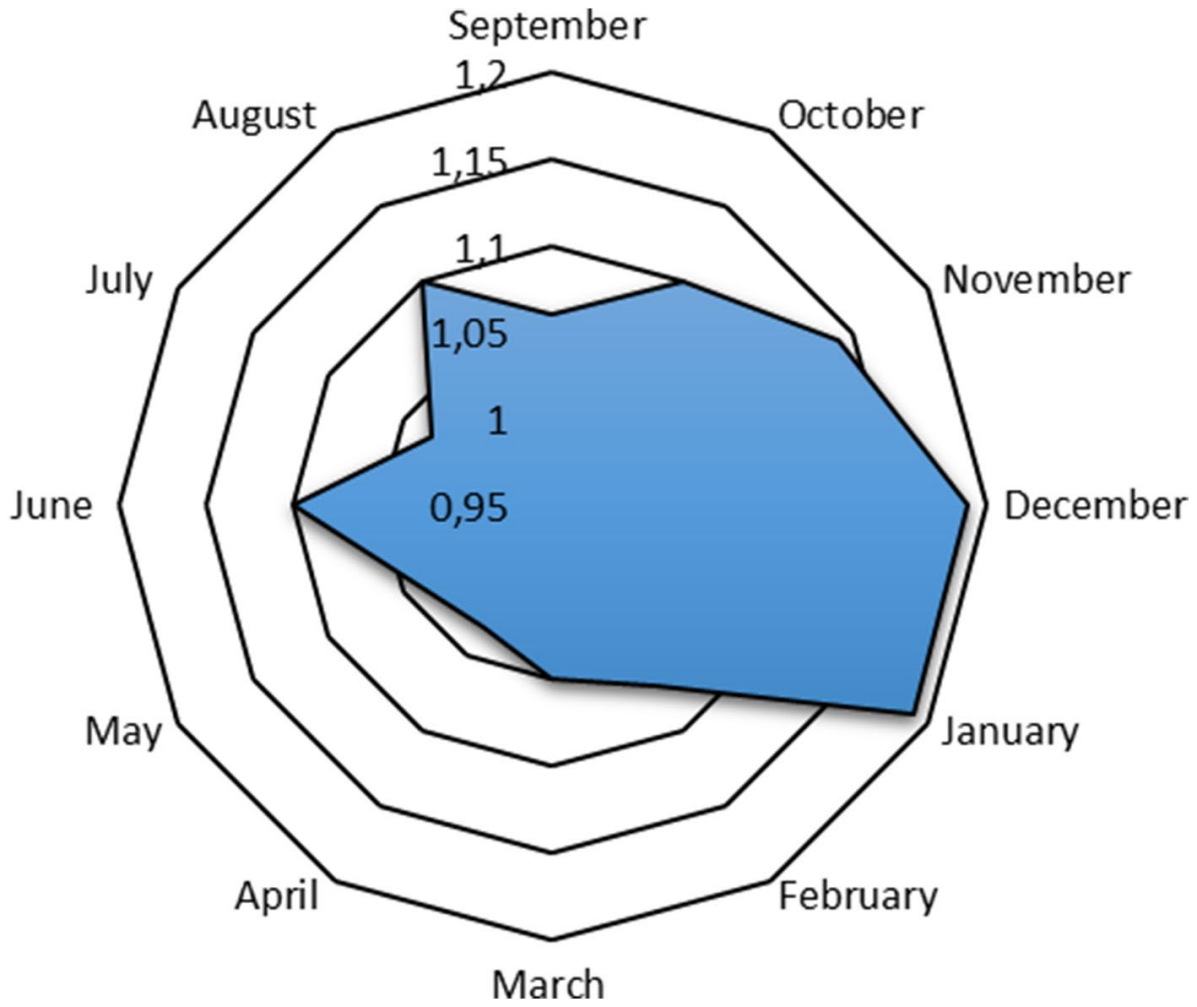
Monthly changes in the∑EAA/∑NEAA ratio of anchovies from the Southeastern Black Sea.

The ∑EAA/∑NEAA ratio is an important indicator of protein quality, with higher values indicating better quality protein (51). In this study, the ∑EAA/∑NEAA ratio for anchovy samples collected monthly varied from 1.03 to 1.19, with significant differences (*p* < 0.05) noted between different months (Figure 5). Both monthly and seasonal ∑EAA/∑NEAA ratios exceeded 1, indicating that anchovy protein is of high quality and provides a well-balanced amino-acid profile as a food source.

## 4 Conclusion

This study provides a comprehensive overview of the changes in proximate composition, fatty acid profile, and amino acid content in European anchovy (*E. encrasicolus*) edible muscle tissue over 12 months. In contrast to previous studies, which typically focused on sampling in specific months or seasons, this study provides detailed information on seasonal changes in nutrient composition throughout the entire year. The results showed that the crude fat content in anchovy muscle tissue collected in the Black Sea was highest during winter, especially in December, January, and February. During this winter period, ∑PUFA/SFA, EPA + DHA, ∑n3 levels, and fatty acid quality indices (FLQ, h/H, and PI) also peaked, with the highest values recorded in January. Protein content did not vary significantly on a monthly or seasonal basis. However, ∑EAA levels and ∑EAA/∑NEAA ratio were highest in winter, especially in December and January. Also, valine, methionine, isoleucine, leucine, phenylalanine, and lysine levels reached their maximum concentrations during the winter season. These findings highlight anchovies as a nutritionally valuable food, particularly due to their high EPA, DHA, and essential amino acid content. The study further demonstrates that nutritional value was greatest in winter, supporting the health benefits of consuming anchovies during this season. From an innovative perspective, this study employs a year-round sampling design, rather than the shorter time frames commonly used in previous research. This approach enables a more reliable evaluation of the seasonal effects on anchovy nutrient composition, providing up-to-date insights into regional nutritional potential. By comparing current results with earlier studies, the research also identifies temporal trends and contributes new knowledge to the literature. Overall, this study fills a critical gap by providing the first continuous, 12-month dataset on anchovy composition in the Southeastern Black Sea, thereby enhancing our understanding of the nutritional value and public health relevance of this key species.

## Data Availability

The original contributions presented in the study are included in the article/supplementary material, further inquiries can be directed to the corresponding author.
